# *PnTgs1-like* expression during reproductive development supports a role for RNA methyltransferases in the aposporous pathway

**DOI:** 10.1186/s12870-014-0297-0

**Published:** 2014-11-18

**Authors:** Lorena A Siena, Juan Pablo A Ortiz, Olivier Leblanc, Silvina Pessino

**Affiliations:** Laboratorio de Biología Molecular, Facultad de Ciencias Agrarias, Universidad Nacional de Rosario, Parque Villarino, (S2125ZAA) Zavalla, Santa Fe, Argentina; Instituto de Botánica del Nordeste -IBONE- (UNNE-CONICET), Facultad de Ciencias Agrarias, Universidad Nacional del Nordeste, Sargento Cabral 2131, 3400 Corrientes, Argentina; Institut de Recherche pour le Développement, ERL 5300 IRD/CNRS, UMR 232 IRD/Université de Montpellier 2, 911 Avenue Agropolis, Montpellier, France

**Keywords:** Apomixis, Apospory, Gene expression, PIMT, RNA processing, Trimethylguanosine synthase

## Abstract

**Background:**

In flowering plants, apomixis (asexual reproduction via seeds) is widely believed to result from failure of key regulators of the sexual female reproductive pathway. In the past few years, both differential display and RNA-seq comparative approaches involving reproductive organs of sexual plants and their apomictic counterparts have yielded extensive lists of candidate genes. Nevertheless, only a limited number of these genes have been functionally characterized, with few clues consequently available for understanding the molecular control of apomixis. We have previously identified several cDNA fragments with high similarity to genes involved in RNA biology and with differential amplification between sexual and apomictic *Paspalum notatum* plants. Here, we report the characterization of one of these candidates, namely, N69 encoding a protein of the S-adenosyl-L-methionine-dependent methyltransferases superfamily. The purpose of this work was to extend the N69 cDNA sequence and to characterize its expression at different developmental stages in both sexual and apomictic individuals.

**Results:**

Molecular characterization of the N69 cDNA revealed homology with genes encoding proteins similar to yeast and mammalian trimethylguanosine synthase/PRIP-interacting proteins. These proteins play a dual role as ERK2-controlled transcriptional coactivators and mediators of sn(o)RNA and telomerase RNA cap trimethylation, and participate in mammals and yeast development. The N69-extended sequence was consequently renamed *PnTgs1-like*. Expression of *PnTgs1-like* during reproductive development was significantly higher in floral organs of sexual genotypes compared with apomicts. This difference was not detected in vegetative tissues. In addition, expression levels in reproductive tissues of several genotypes were negatively correlated with facultative apomixis rates. Moreover, *in situ* hybridization observations revealed that *PnTgs1-like* expression is relatively higher in ovules of sexual plants throughout development, from premeiosis to maturity. Tissues where differential expression is detected include nucellar cells, the site of aposporous initials differentiation in apomictic genotypes.

**Conclusions:**

Our results indicate that *PnTgs1-like* (formerly N69) encodes a trimethylguanosine synthase-like protein whose function in mammals and yeast is critical for development, including reproduction. Our findings also suggest a pivotal role for this candidate gene in nucellar cell fate, as its diminished expression is correlated with initiation of the apomictic pathway in plants.

**Electronic supplementary material:**

The online version of this article (doi:10.1186/s12870-014-0297-0) contains supplementary material, which is available to authorized users.

## Background

Gametophytic apomixis in flowering plants refers to asexual reproduction through seeds [1]. This reproductive mode can be achieved through diverse paths [2] and is widespread in angiosperms [3]. With two major distinctions, the developmental programs governing plant sexuality typically form the basis of apomixis. The first important difference involves differentiation of one or more functional unreduced female gametophytes, which occurs within the nucellus after either meiosis failure (diplosporous type) or nucellar cell fate alteration (aposporous type). Second, female gamete fertilization is not required for seed formation, leading to the development of a maternal embryo by parthenogenesis. The endosperm originates autonomously or after fertilization of the polar nuclei (pseudogamy).

*Paspalum* [4], one of the largest genera within Poaceae (Gramineae), encompasses approximately 370 species classified into four subgenera (*Anachyris*, *Ceresia*, *Harpostachys*, and *Paspalum sensu stricto*) (reviewed in [5]). *Paspalum notatum* is a member of the subgenus *Paspalum* and forms an agamic complex comprising self-sterile sexual diploids and self-fertile apomictic autotetraploids [5]. *Paspalum notatum* apomictic genotypes reproduce through apospory. In immature ovules at the premeiotic stage, one to several companion nucellar cells surrounding the megaspore mother cell enlarge, undergo a series of mitoses, and finally differentiate into non-reduced embryo sacs termed aposporous embryo sacs (AESs). AESs may coexist with the single meiotically-derived embryo sac (MES), or alternatively outcompete it and occupy the entire volume of the mature ovule [5]. The structure of a *Paspalum notatum* MES is typical of Gramineae species: one egg cell, two synergids, one bi-nucleated central cell, and a mass of proliferating antipodals [6]. On the other hand, AESs exhibit a distinct pentanucleate morphological structure (*Paspalum*-type embryo sacs) characterized by the presence of one egg cell, two synergids, and one bi-nucleated central cell but no antipodals; this structure allows straightforward classification by cytoembryological analysis of clarified ovules [7]. At anthesis, MESs undergo typical angiosperm double fertilization to produce viable seeds. In contrast, AESs usually develop into seeds after fertilization of the central cell with parthenogenetic embryo formation (pseudogamy). Nearly 100% of ovules of obligate aposporous individuals contain only AESs, whereas facultative aposporous individuals display variable proportions of mature mixed ovules that include both a MES and one or more AES. Although fully sexual polyploid individuals do not exist in nature, some genotypes have been produced artificially through colchicine-induced chromosome doubling of sexual diploids [8] or by crossing facultative apomicts [9].

Apomixis in *Paspalum notatum* is inherited as a dominant, monogenic trait, with a distorted segregation ratio, and is associated with a single genomic region, the Apospory Controlling Region (ACR) [7,10-13]. Mapping approaches have revealed strong suppression of recombination within the *Paspalum notatum* ACR and disomic inheritance, whereas the remaining genomic regions show polysomic inheritance [12,13]. Partial resolution of the ACR has unveiled a rather complex genomic structure comprising genomic sectors syntenic to rice chromosomes 2 and 12 but extensively rearranged through inversion, translocation, and/or insertion of low- and high-copy number retroelements [10,11,13-15]. These features are strongly consistent with the lack of recombination detected by genetic mapping and the distorted segregation ratios against apospory observed in some progenies. Whether the peculiar ACR genomic structure is pivotal to the transcriptomic changes required to switch from sexuality to apospory is nevertheless unknown. Interestingly, our current knowledge suggests that although gene-poor, the ACR contains several sequences putatively encoding proteins, including an MT-A70-like candidate (mRNA N6-adenosine-methyltransferase) [14]. This enzyme catalyzes N6-adenosine methylation in nascent mRNA and plays key roles in cell fate decision in multiple eukaryote systems [16]. In particular, MT-A70 loss of function in the model plant *Arabidopsis thaliana* leads to early embryo development failure [17]. Another interesting sequence mapped onto the ACR is a K homology (KH) domain-containing protein, which is an RNA-binding protein implicated in mRNA stability and regulation of gene expression at the post-transcriptional level [18,19]. KH proteins have been associated with maintenance of an inactive chromatin state in *knox* genes located within the peripheral zone of the shoot apical meristem required for proper leaf development in maize [20].

Transcriptomic surveys have enabled the identification of numerous candidate genes associated with aposporous apomixis in plant species such as *Brachiaria brizantha*, *Poa pratensis*, *Paspalum notatum*, *Panicum maximum*, *Boechera* spp., *Paspalum simplex* and *Hieracium* spp. [21-27]. Comparative transcriptomic analysis of *Paspalum notatum* has suffered from several major drawbacks commonly found in apomictic systems: lack of genuine near-isogenic apomictic and sexual lines, high heterozygosity, and limited genomic resources (reviewed in [5]). RNA profiling assays based on differential display have been designed to overcome these difficulties and have been used to identify a set of 45 candidates usually down-regulated during apomictic development compared with sexual formation [23,28]. Interestingly, two of these genes (N4 and N69) show significant similarity to genes encoding RNA methyltransferases [23].

The identification of an RNA-N6-adenosine-methyltransferase gene and an RNA-binding protein gene within the *Paspalum notatum* ACR, coupled with two additional RNA methyltransferases differentially expressed in flowers of sexual and apomictic plants, prompted us to explore the possible role of RNA methylation in aposporous reproductive development. As part of this effort, we sought to infer the possible function of the N69 cDNA candidate in reproductive development. To accomplish this goal, we characterized the candidate, identified its putative orthologs in model species, and analyzed its expression in reproductive tissues of sexual and apomictic genotypes at key developmental steps.

## Results

### N69 sequence characterization

**Figure 1:**
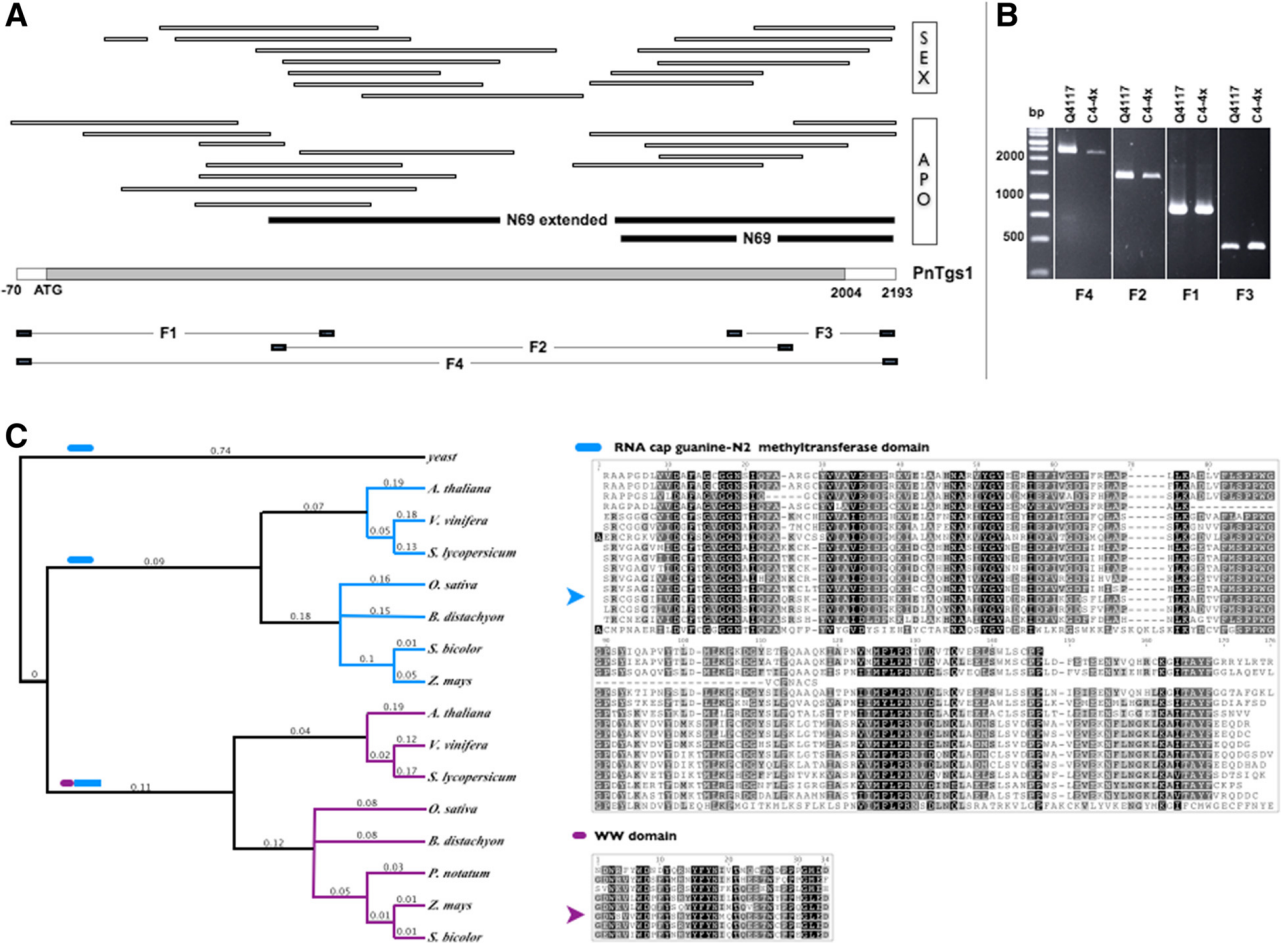
**N69 partial cDNA derives from a plant-specific gene encoding a trimethyl guanosine synthase. (A)** A consensus cDNA sequence was assembled using N69 cDNA, a N69-extended fragment obtained by RACE (black bars) and reads from RNA 454 sequencing in sexual (C4-4x) and apomictic (Q4117) reproductive tissues (white bars). This sequence contains a 2004 bp predicted ORF coding for a product sharing homology with yeast TRIMETHYL GUANOSINE SYNTHASE 1 (TGS1). **(B)** Primers were designed in order to amplify 4 different sequence fragments (F1, F2, F3, F4), which were amplified and sequenced for validation, from reproductive tissues of apomictic (Q4117) and sexual (C4-4x) genotypes. **(C)** Phylogenetic analysis of proteins similar to yeast TGS1 from several yeast and plant model species revealed that the *Paspalum notatum* predicted product is highly similar with plant specific members of the TGS1 protein family defined by the association of the RNA-cap guanine-N2 methyltransferase domain with a WW domain. Domains are indicated as colored ticks. ClustalW alignments are shown on the right (arrows indicate *Pasalum notatum* sequences).

The deduced amino acid sequence of the 888-nucleotide N69 cDNA fragment originally isolated from *Paspalum notatum* [23] showed strong homology with the RNA-cap guanine-N2 methyltransferase domain (PF09445; *E*-value: 1.4 × 10^−46^) [23]. In yeast, mammalian, and *Drosophila* genomes, PF09445 is encoded by a single gene, TGS1 (trimethylguanosime synthase), a conserved nucleolar methyl transferase responsible for the conversion of m(7)G cap of sn-, sno- and telomerase RNAs to m(2,2,7)G, as well as for nucleolar assembly and splicing of meiotic pre-mRNAs [29-31]. On the other hand, plant genomes contain two different genes encoding TGS1-like proteins. The first one, that is conserved across Eukaryotes, includes only the RNA-cap guanine-N2 methyltransferase domain (GRMZM2G151887, OS06T0187100, and AT1G30550, respectively in maize, rice and *A. thaliana*) while the second one displays a plant-specific architecture, with the RNA methyltransferase domain associated with a WW domain involved in protein-protein interactions (GRMZM2G347808, OS03T0396900 and AT1G45231, respectively in maize, rice and *A. thaliana*). By taking advantage of Roche 454 RNA sequencing data obtained from reproductive tissues of sexual and apomictic plants, which we combined with RACE experiments using Marathon cDNA libraries, we assembled a single contig representing the N69-extended cDNA consensus sequence covering the whole N69 CDS (see Methods and Figure 1). BLAST analyses further demonstrated that it derived from transcripts of a *Paspalum notatum* gene homologous to a plant specific member (WW + AdoMet donains) of theTGS1 family, that we consequently named *PnTgs1-like*. Note that the N69-extended sequence contains the complete CDS of *PnTgs1-like* but that we could not resolve the full 5′ and 3′ UTRs.

Using this information, we designed a set of primers to amplify and sequence both overlapping fragments (F1, F2, F3) and the complete sequence (F4) from mRNA samples extracted from flowers of Q4117 (apomictic) and C4-4x (sexual) plants (Figure 1; Table 1). We recovered two complete sequences (Apo*PnTgs1* and Sex*PnTgs1*) with synonymous variant sites, including SNPs and a few INDELs (deposited in GeneBank under accession numbers BankIt1742582 Apo*PnTgs1* KM114904 and BankIt1742582 Sex*PnTgs1* KM114905) but sharing 96.9% identity at the amino acid level, thus suggesting that PnTGS1*-like* is functional in both sexual and apomictic plants. The construction of a phylogenetic tree including 16 total TGS1-like protein sequences originated from yeast and plants revealed a higher similarity with plant-specific sequences, containing both WW + AdoMet domains (Figure 1).

**Table 1:** **Primers used to recover partial and complete** ***PnTgs1-like*** **sequences from apomictic and sexual genotypes (fragments F1, F2, F3, F4)**

**Primer**	**Sequence**	**Tm (°C)**
F1 forward	5′-AAACCCAAACGGCTAAAACC-3′	60.2°C
F1 reverse	5′-CCTTGACGTGTCCTCAGACTCTAGC-3′	64.9°C
F2 forward	5′-TCTGCTGAAAAAGCCCCTGGC-3′	51.2°C
F2 reverse	5′-CCTCAACCGCCCATGGAGGA-3′	66°C
F3 forward	5′-CAGGAGTTACAGATTTGGCCCTTGC-3′	67.8°C
F3 reverse	5′-GGGGTGGACCTGACTATGCTAAAG-3′	66.3°C
F4 forward	5′-AAACCCAAACGGCTAAAACC-3′	60.2°C
F4 reverse	5′-GGGGTGGACCTGACTATGCTAAAG-3′	66.3°C

### Correlation of *PnTgs1-like* expression with reproductive behaviors

**Figure 2:**
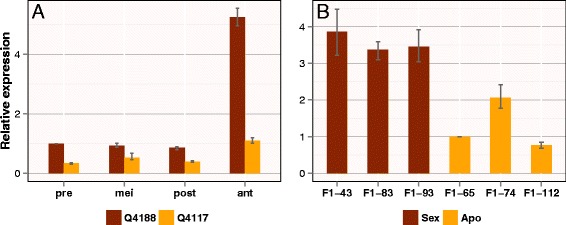
**Quantitative real-time PCR analysis of** ***PnTgs1-like*** **transcripts in sexual and apomictic** ***Paspalum notatum*** **plants. (A)** Flowers were collected at premeiosis (pre), meiosis (mei), postmeiosis (post), and anthesis (ant) from Q4188 (sexual) and Q4117 (apomictic). **(B)** Expression was measured in three sexual and three apomictic individuals of an F_1_ population derived from Q4188 × Q4117 [12,13].

We first measured *PnTgs1-like* expression levels during reproductive development of apomictic (Q4117) and sexual (Q4188) plants using RNA samples extracted from spikelets collected at premeiosis, meiosis, postmeiosis, and anthesis. As shown in Figure 2A, quantitative analysis revealed significantly higher expression levels in the sexual genotype at all developmental stages. In addition, expression in the sexual genotype at anthesis was increased around 5-fold compared with that measured at premeiosis. Such an increase was not observed in the apomictic genotype.

To determine whether this differential expression was genuinely associated with the reproductive mode, we investigated *PnTgs1-like* expression in spikelets collected at anthesis from three sexual and three apomictic F_1_ plants derived from a Q4188 × Q4117 cross. All genotypes showed *PnTgs1-like* expression levels similar to those observed in the corresponding sexual and apomictic progenitors (Figure 2B).

**Figure 3:**
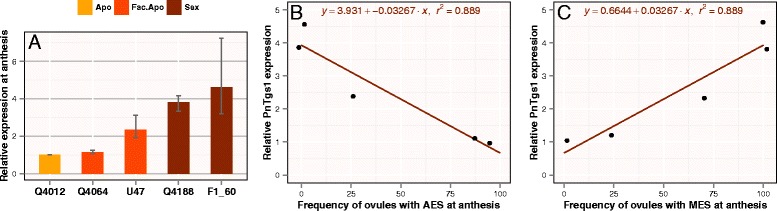
**Correlation of** ***PnTgs1-like*** **expression at anthesis with reproductive behaviors. (A)** Relative quantitative expression in sexual, facultative apomictic, and fully apomictic plants categorized according to the percentage of ovules carrying meiotic and aposporous embryo sacs (MES and AES, respectively) as shown in Table 2. *PnTgs1* expression was oppositely correlated with the percentage of ovules carrying AESs **(B)** compared with MESs **(C)**. B and C show plots of fitted values from linear regressions obtained using the *lm* command of the R program. Error bars indicate ranges of qPCR replicates

We next verified whether *PnTgs1-like* expression levels were correlated with facultative apomixis rates previously recorded for *Paspalum notatum*. For this purpose, we used several genotypes whose apospory/sexuality expression ratios have been reported by Quarin et al. [9], Stein et al. [12], and Espinoza et al. [32] (Table 2). Interestingly, the expression level of *PnTgs1-like* increased with the degree of sexuality of the tested genotypes (Figure 3A). Moreover, we detected a positive correlation between *PnTgs1-like* expression levels and the percentage of ovules carrying MESs (R^2^ = 0.889); conversely, expression levels were negatively correlated with the percentage of ovaries carrying AESs (R^2^ = 0.889) (Figure 3B and C).

**Table 2:** ***PnTgs1*** **representation in** ***Paspalum notatum*** **genotypes with variable levels of apospory expression**

**Plant**	**% OMES**	**%OAES**	***PnTgs1*** **relative representation** ^**a**^	**Standard error** ^**b**^	**Results** ^**c**^	**References** ^**d**^
Q4012	0.0	100	1.000	-	-	[32]
Q4064	17.6	78.4	1.151	1,058 - 1,252	UP	[32]
U47	63.8	76.6	2.339	1,916 - 3,129	UP	[32]
Q4188	94	0	3.807	3,533 – 4,153	UP	[9]
F_1_-60	100	0	4.616	3,210 - 7,229	UP	[12]

% OMES: Percentage of ovules carrying meiotic embryo sacs; % OAES: percentage of ovules carrying aposporous embryo sacs (as indicated in the references quoted in the last column). The percentages of ovules carrying meiotic or aposporous embryo sacs do not sum up to 100% because mixed ovules with both embryo sac types occurred frequently.

^a^Relative representation of the *PnTgs1* transcript in floral tissues as estimated by real-time PCR. Genotype Q4012 (full apomictic, 0.0% OMES, relative expression: 1.000) was used as a control in REST-RG (Corbett Life Sciences) expression analysis.

^b^Standard error as calculated by REST-RG software.

^c^Results indicating up-regulation (UP) or down-regulation (DOWN) at a highly significant level as informed by REST-RG.

^d^Studies reporting % OMES and % OAES corresponding to the different genotypes analyzed here.

Finally, we detected similar *PnTgs1-like* expression levels in vegetative tissues (leaves and roots) of sexual and apomictic plants (Additional file 1). *PnTgs1*expression was detectable at levels that were lower but did not differ significantly from those measured in floral tissues. These results indicate that this gene might be performing a common role in non-reproductive tissues of both apomictic and sexual plants, but display a specific function in floral tissues associated with the reproductive mode.

### *In situ* localization of *PnTgs1-like* expression

Using *in situ* mRNA hybridization, we investigated *PnTgs1-like* expression in *Paspalum notatum* spikelets sampled at two developmental stages critical to the success of apospory, late premeiosis/meiosis and anthesis, respectively concomitant with aposporous initial differentiation and embryo parthenogenesis.

**Figure 4:**
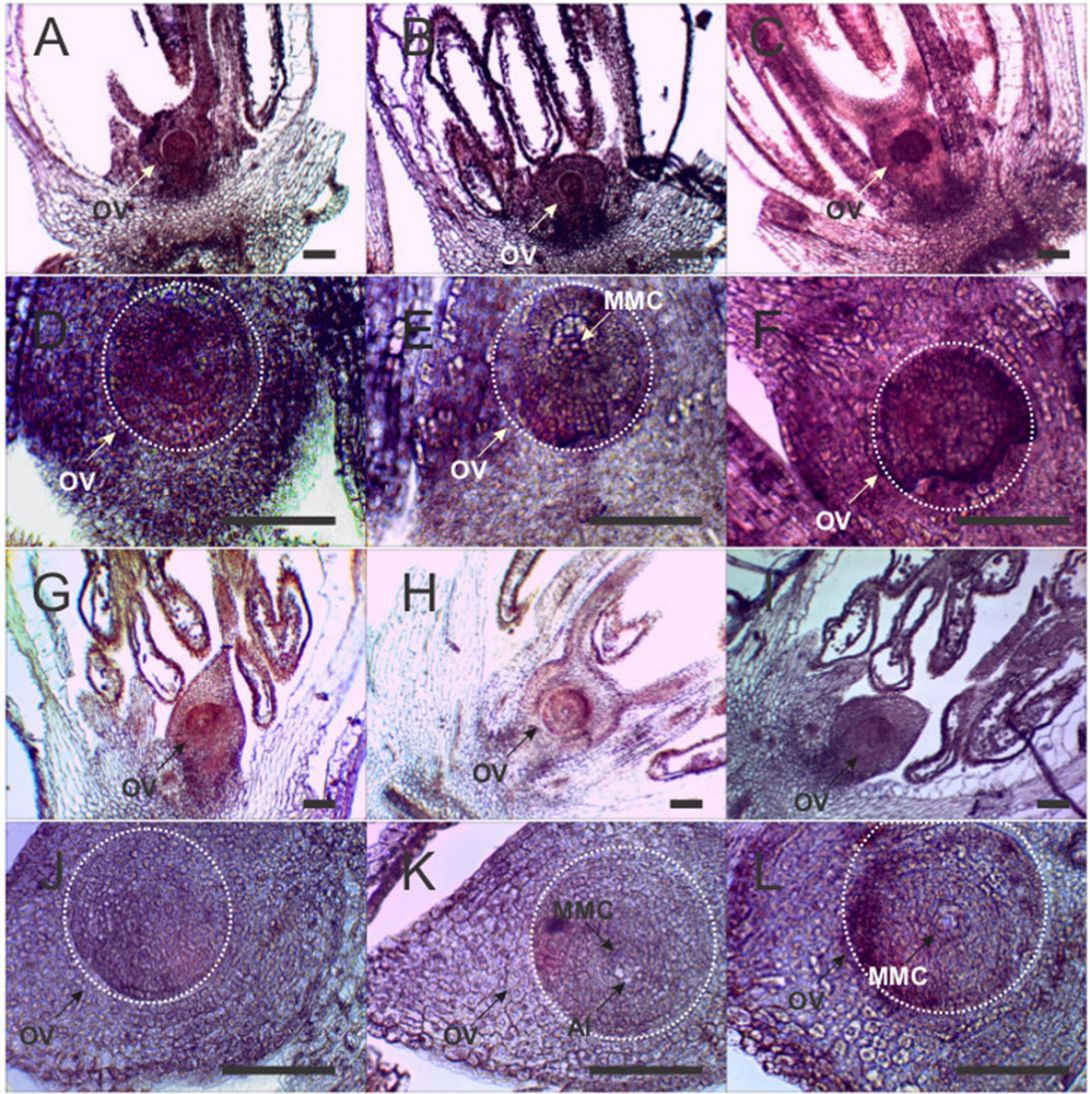
***In situ*** **expression of** ***PnTgs1-like*** **in reproductive tissues of sexual (Q4188) and apomictic (Q4117)** ***Paspalum notatum*** **genotypes at late premeiosis/meiosis.** Sexual genotype Q4188 reproductive tissues (ovaries + anthers) hybridized with an antisense *PnTgs1-like* RNA probe, showing expression mainly in the ovule **(A, B, C)**. Larger images of ovules originated from sexual genotype Q4188 **(D, E, F)**. Apomictic genotype Q4117 reproductive tissues (ovaries + anthers) displaying a lower expression of *PnTgs1-like* **(G, H, I)**. Larger images of ovules originated from apomictic genotype Q4117 **(J, K, L)**. White dotted lines indicate ovule boundaries. OV: ovule; MMC: megaspore mother cell; AI: apospory initials. Bars: 50 μm.

**Figure 5:**
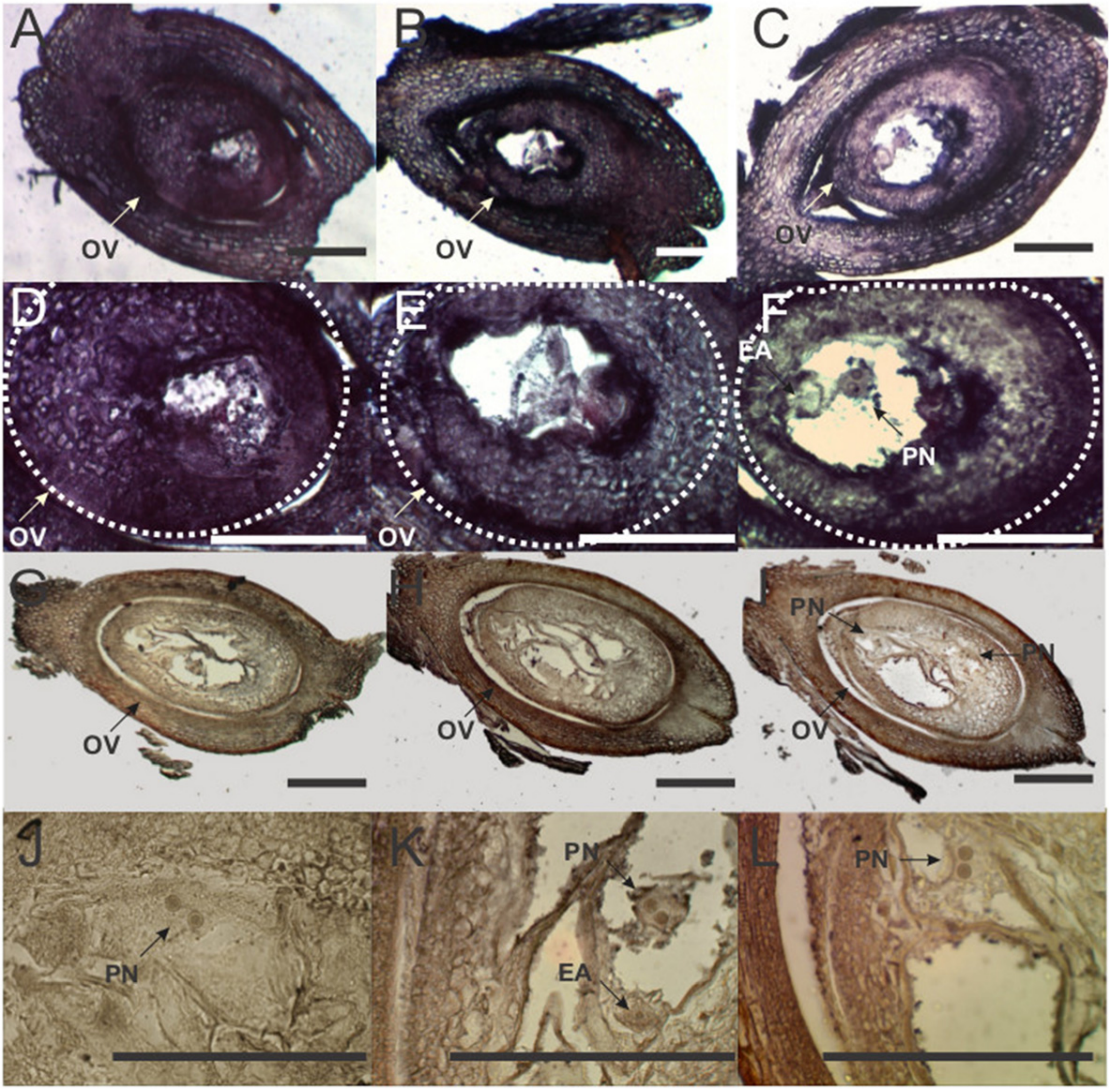
***In situ*** **expression of** ***PnTgs1-like*** **in reproductive tissues of sexual (Q4188) and apomictic (Q4117)** ***Paspalum notatum*** **genotypes at anthesis.** Sexual genotype Q4188 ovaries hybridized with an antisense *PnTgs1-like* RNA probe, showing an intense signal in nucella and integuments **(A, B, C)**. Larger images of ovules originated from sexual genotype Q4188 **(D, E, F)**. Apomictic genotype’s (Q4117) ovaries displaying a lower expression of *PnTgs1-like* and showing several non-reduced embryo sacs within the ovule **(G, H, I)**. Details of embryo sacs originated from apomictic genotype Q4117 **(J, K, L)**. White dotted lines indicate ovule boundaries. OV: ovule; PN: polar nuclei; EA: egg apparatus. Bars: 100 μm.

During late premeiosis/meiosis, a strong signal was observed in nucellar cells and anther tapetum of the sexual genotype. Fainter signals at similar locations were detected in the apomictic genotype (Figure 4). The same expression trend was observed at anthesis: both nucellar and integumentary tissues displayed an intense signal in the sexual genotype. On the other hand, almost no signal was detected in the apomictic genotype (Figure 5).

These results indicate that *PnTgs1-like* is active in the nucellus of sexual *Paspalum notatum* plants from early developmental stages until maturity, whereas expression is strongly reduced throughout reproductive development in apomictic genotypes.

## Discussion

The N69 cDNA fragment was initially recovered during screening to identify transcriptional differences between spikelets collected from sexual and apomictic *Paspalum notatum* plants [23]. Subsequent sequence analysis revealed that this fragment belongs to a gene encoding PIMT (PRIP-interacting protein with methyltransferase domain)/TGS1, a methyltransferase involved in sn(o)RNA biogenesis, mRNA splicing, and coactivation of PPAR (peroxisome proliferator activated receptor)-regulated gene expression (reviewed in [33]).

PIMT/TGS1, first isolated as a transcriptional co-activator PRIP-interacting protein from *Saccharomyces cerevisiae* [34], has been extensively studied in yeast, flies, and mammals (reviewed in [33]) but remains poorly characterized in plants. Interestingly, all eukaryotes possess a single *tgs1* copy—except for plant genomes, which usually carry at least two copies. PIMT/TGS1 typically contains a methyltransferase domain and two binding domains; this structure allows interactions with RNA and S-adenosyl-L-methionine, the methyl donor in the methyl transfer reaction [34]. The post-transcriptional conversion of 7-methylguanosine caps (m^7^G) into 2,2,7-trimethylguanosine (m_3_G) catalyzed by PIMT/TGS1 plays a central role in the biogenesis of sn(o)RNAs and telomerase RNAs [30,35]. In addition, PIMT/TGS1 is pivotal for transcriptional modulation in several contexts. It interacts with and co-localizes to the nucleus along with histone acetyl transferase (HAT)-containing transcriptional coactivators such as CBP/Ep300 and non-HAT-containing coactivators such as the Mediator subunit Med1 (PPAR binding protein; PBP/TRAP220/DRIP205) and PRIP [34,36,37]. PIMT has been proposed to serve as a molecular bridge between HAT- and non-HAT-containing transcriptional complexes and to control nuclear receptor-mediated transcription. Moreover, ERK2 phosphorylation at Ser^298^ of PIMT/TGS1 activates transcriptional activity at some promoters, suggesting a direct role for signal transduction pathways in modulating transcription [38].

The abolishment of PIMT/TGS1 function causes a wide range of phenotypic defects in different eukaryotic non-plant model systems. In *S. cerevisiae*, these alterations consist of cold-sensitive splicing defects, growth delay at low temperatures, loss of nucleolar structural organization, pre-rRNA processing deficiency, and meiotic failure after aberrant splicing of key regulators [29,31,35]. In mammals, PIMT/TGS1 loss of function leads to alteration of cell cycle progression, embryo lethality, and increased hepatic gluconeogenesis [38-40]. In *Drosophila*, embryo lethality in the early pupal stage has been reported [41].

Interestingly, comparative transcriptomic analyses of sexual vs. apomictic reproduction in plants, including *Paspalum notatum*, have provided sets of modulated genes enriched in ontological families strongly associated with PIMT/TGS1 function in eukaryotes. A major set consists of ribosomal RNAs and ribosomal protein genes [22,23,27], an observation consistent with the role of PIMT/TGS1 in both nucleolar organization and pre-rRNA processing [29]. Other TGS1-related functional classes that are differentially expressed in sexual and apomictic plants are proteasome-related proteins, cytoskeletal proteins, and ERK2 cascade member genes [22,23,27]. With regards to the body of data collected in non-plant species for TGS1 function, our observations for reduced expression of *PnTgs1-like* during female reproductive development in apomictic genotypes suggests that the transition from sexuality to aposporous development might depend on alterations of the splicing-machinery operating in conjunction with ERK2-mediated transcriptional regulation. Such differential expression was not observed in vegetative tissues. Whether a reduction of *PnTgs1-like* expression is causal for apomixis and which mechanisms are responsible for the differential expression in reproductive tissues definitively require further investigations, including trimethylguanosine synthase activity assays, RNA methylation profiling, and mutant analysis in model plant species.

Comparative transcriptional analyses and mutant characterization have revealed that apomictic developments likely emerged after alterations in the prevailing transcriptional dynamics of reproductive tissues or cells of sexual plants [42]. In particular, expression patterns in female reproductive tissues or cells are modulated during development by specific RNA-dependent DNA methylation pathways and, interestingly, some chromatin-remodeling enzymes participating in these pathways are down-regulated in maize-*Tripsacum* apomictic hybrids; their loss of function in maize causes profound reshaping of transcriptional activities and developmental heterochronicity partially mimicking apomictic developments [43,44]. Defining the nature of the sequences targeted by these silencing pathways is critical to resolve two issues: the precise identification of these pathways’ roles in the evolution of apomictic reproduction from sexuality, and the elucidation of the mechanisms responsible for their chronological and spatial inhibition. With regard to the latter question, we believe that the function of TGS1 in both RNA biology and transcriptional pattern regulation offers the basis for an attractive model involving altered RNA processing to explain coordinated loss of function of key regulators.

## Conclusions

Our results indicate that *PnTgs1-like* shows high expression in nucellar cells of sexual plants while its representation is significantly reduced in aposporous plants. Moreover, *PnTgs1* expression levels are negatively correlated with the occurrence of AESs. At the same time, genes usually associated with PIMT function (such as rRNA and ribosomal protein genes) have been reported by our research group [23] and others [22,27] to be differentially expressed between sexual and apomictic plants. These findings suggest that PnTGS1-like may participate in the repression of AES formation in nucellar cells surrounding the legitimate germ cell lineage. To confirm our hypothesis of a pivotal role for methylation-dependent RNA processing in the emergence of asexual reproductive pathways, we are currently using a transformation platform recently established in our laboratory [45] to assess the effects of the plant specific TGS1-like protein on the reproductive development of plant model species and *Paspalum notatum*.

## Methods

### Plant material

The following tetraploid (2*n* =4*x* =40) *Paspalum notatum* genotypes were used in this study: i) fully apomictic genotypes Q4117 and Q4012 [32,46]; ii) facultative apomictic genotypes Q4064 and U47 [32]; iii) fully sexual genotypes C4-4x and Q4188 [8,9]; and iv) three fully sexual (#43, #60, #93) and three fully apomictic (#40, #65, #74) F_1_ hybrids derived from a Q4188 × Q4117 cross [12,13]. The *Paspalum notatum* genotypes were obtained from the IBONE live germplasm collection (Instituto de Botánica del Nordeste, IBONE-CONICET, Argentina).

### cDNA sequencing

RACE experiments [47] were conducted, following the manufacturer’s instructions, on two cDNA Marathon libraries (Clontech, Mountain View, California, USA) produced from Q4117 and Q4188 mRNA samples extracted from spikelets during late premeiosis/meiosis (developmental stages I/II of Laspina et al. [23]). Primers were designed with Primer 3 Plus (http://www.bioinformatics.nl/cgi-bin/primer3plus/primer3plus.cgi/) [48]. PCR amplifications were performed using a DNA Engine Peltier thermal cycler (Bio-Rad, Hercules, California, USA) in 25-μl reactions containing tricine buffer (0.01 M tricine and 0.1 mM EDTA) supplemented with 1 μl of a 1:250 Marathon library dilution, 0.2 μM of each specific primer, 0.2 μM AP1 primer (matching the Marathon adapter AP1), 1× Taq-LOADTM Mastermix (MP Biomedicals), 1.5 U *Taq* polymerase (Promega, Madison, Wisconsin, USA), 1.5 mM MgCl_2_, and 200 pM dNTPs. Amplicons were electrophoresed on 1.5% (w/v) agarose gels and stained with 1% (v/v) ethidium bromide. Fragments of interest were purified with a QIAquick gel extraction kit (Qiagen, Valencia, California, USA), cloned into a p-GEM-T Easy vector (Promega), and transferred by thermal shock into *Escherichia coli* DH5α TOPO competent strands (Invitrogen/Life Technologies, Carlsbad, California, USA). Plasmids were purified using a QIAprep Spin Miniprep kit (Qiagen), and the amplified RACE fragments were sent to Beckman Coulter Genomics (London, UK) for sequencing.

For pyrosequencing, Q4117 (apomictic) and C4-4x (sexual) total RNA samples were extracted from balanced bulks of spikelets collected at premeiosis, meiosis, postmeiosis, and anthesis. mRNA enrichment, library preparation, emulsion PCR, 454 Genome Sequencer FLX + (Roche, Penzberg, Germany) sequencing, and bioinformatic analysis were performed by INDEAR (Instituto de Agrobiotecnología de Rosario, Rosario, Argentina).

### Sequence analyses

The RACE fragment sequence was trimmed using the VecScreen algorithm at NCBI (www.ncbi.nlm.nih.gov), and the primer sequences were eliminated using Sequencher 4.1.4 (Gene Codes Corporation). The *PnTgs1-like* contig was then assembled in Sequencher from the RACE extended N69 sequence and the Roche 454 transcripts. BLASTN and BLASTX similarity analyses were conducted using NCBI (www.ncbi.nlm.nih.gov) and Gramene database (http://ensembl.gramene.org/genome_browser/index.html). Alignments were performed using Clustal Omega at the EBI-EMBL website (http://www.ebi.ac.uk/Tools/msa/clustalo/). Open reading frames were located with ORF finder (http://www.ncbi.nlm.nih.gov/gorf/), and translation was carried out using the ExPASy translate tool (http://web.expasy.org/translate/).

### Quantitative real-time PCR

Total RNA was extracted from spikelets collected at several developmental stages (premeiosis, meiosis, postmeiosis, and anthesis) using an SV Total RNA isolation kit (Promega). cDNAs were synthesized from 1 μg of total RNA using Superscript II retrotranscriptase (Invitrogen/Life Technologies). Quantitative real-time PCR amplifications were performed in 25-μl final reaction volumes containing 200 nM gene-specific primers (N69F1 and N69R1; Table 1), 1× qPCR Real Mix (Biodynamics, Buenos Aires, Argentina), and 20 ng cDNA. Amplifications were performed in a Rotor-Gene Q thermocycler (Qiagen) programmed as follows: 2 min at 94°C followed by 45 cycles of 15 s at 94°C, 30 s at 55°C, and 40 s at 72°C. A melting curve (10-s cycles from 72 to 95°C, with the temperature increased by 0.2°C per cycle) was produced at the end of the cycling period. Quantitative real-time PCRs were performed in triplicate from cDNAs obtained from two biological replicates. Values were normalized using β-tubulin as an internal reference gene, since in former work this gene was reported to show a stable representation in flowers of sexual and apomictic plants of the same ploidy level in *Poa pratensis* and *Paspalum notatum* [22,49,50]. Relative expression levels were calculated using REST-RG software (Corbett Life Sciences).

### *In situ* hybridization experiments

Spikelets of sexual (Q4188) and apomictic (Q4117) *Paspalum notatum* genotypes were collected and fixed in a solution of 4% paraformaldehyde/0.25% glutaraldehyde/0.01 M phosphate buffer (pH 7.2), dehydrated in an ethanol/xylol series, and embedded in paraffin. Specimens were sliced into 10-μm-thin sections and placed onto slides treated with 100 μg ml^−1^ poly-L-lysine. Paraffin was removed with a xylol/ethanol series. Both sense (T7) and antisense (SP6) RNA probes were produced using a plasmid containing the N69 5′ RACE clone. Probes were labeled using a Roche DIG RNA labeling kit (SP6/T7) and hydrolyzed into 150–200-bp fragments. Following prehybridization at 37°C for 10 min in 0.05 M Tris–HCl buffer (pH 7.5) containing 1 μg ml^−1^ proteinase K, hybridization at 37°C was performed overnight in a buffer containing 10 mM Tris–HCl (pH 7.5), 300 mM NaCl, 50% deionized formamide, 1 mM EDTA (pH 8), 1× Denhardt’s solution, 10% dextran sulfate, 600 ng ml^−1^ total RNA, and 60 ng of the corresponding probe. Detection was performed following the Roche DIG detection kit instructions using anti-DIG AP and NBT/BCIP as substrates.

### Availability of supporting data

The data set supporting the results of this study is included within the manuscript and its additional file(s).

## Additional file

Additional file 1: Figure S1. Real-time PCR analysis of *PnTgs1* expression in leaves and roots. Description of data: Expression was detected in both leaves and roots, but no significant difference was observed between apomictic and sexual genotypes. On the contrary, differential expression was observed in flowers. Error bars indicate ranges of qPCR replicates.
