# Supplementary figures and images for: PnTgs1-like expression during reproductive development supports a role for RNA methyltransferases in the aposporous pathway
Source: BMC Plant Biol. 2014 Nov 18;14:297. doi: 10.1186/s12870-014-0297-0 (PMC4243328; doi:10.1186/s12870-014-0297-0)

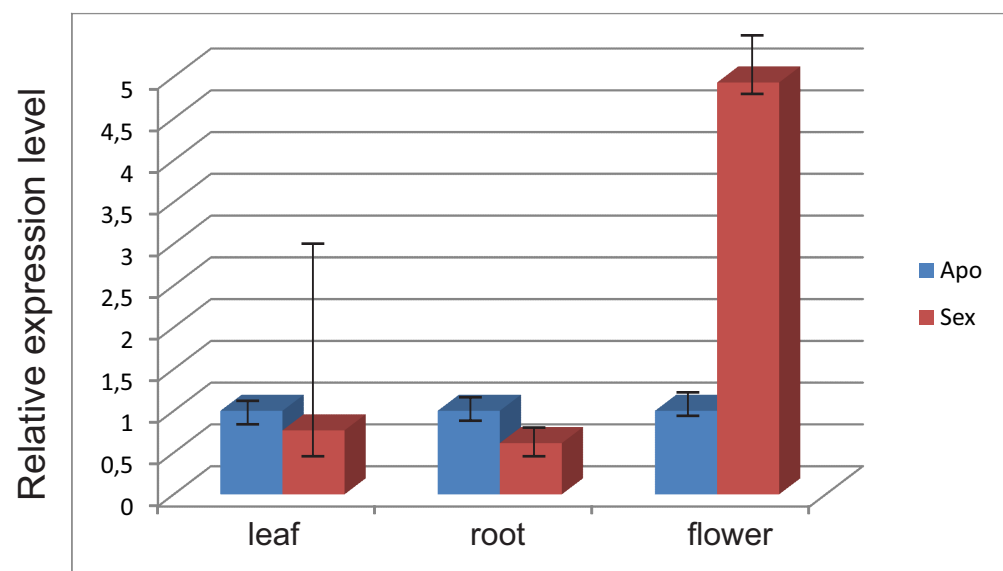

Supplement: Additional file 1: Figure S1. — Real-time PCR analysis of PnTgs1 expression in leaves and roots. Description of data: Expression was detected in both leaves and roots, but no significant difference was observed between apomictic and sexual genotypes. On the contrary, differential expression was observed in flowers. Error bars indicate ranges of qPCR replicates. [file 12870_2014_297_MOESM1_ESM.pdf]
